# Relaxor Ferroelectric Polymers: Insight into High Electrical Energy Storage Properties from a Molecular Perspective

**DOI:** 10.1002/smsc.202000061

**Published:** 2021-02-15

**Authors:** Yang Liu, Yen-Ting Lin, Aziguli Haibibu, Wenhan Xu, Yao Zhou, Li Li, Seong H. Kim, Qing Wang

**Affiliations:** ^1^ Department of Materials Science and Engineering The Pennsylvania State University University Park PA 16802 USA; ^2^ Department of Chemical Engineering The Pennsylvania State University University Park PA 16802 USA

**Keywords:** dielectric properties, electrical energy storage, ferroelectric polymers, phase transition in relaxor polymers, scanning probe microscopy

## Abstract

Relaxor ferroelectric polymers exhibit both high dielectric constants and low remnant polarization and thus deliver much higher energy densities and greater charge–discharge efficiencies than normal ferroelectrics for capacitive energy storage applications. Herein, dielectric energy storage behavior of several newly discovered relaxor ferroelectric polymers is studied from a molecular perspective. It is observed that the homopolymers exhibit very slim polarization–electric field loops and the highest charge–discharge efficiencies among ferroelectric polymers, which are attributed to the highly disordered chain conformation as evidenced from the scanning probe microscopy results. Based on the findings on the relaxor homopolymers, the benchmark relaxor ferroelectric terpolymers is revisited and insights into their outstanding capacitive performance are provided. Moreover, it is found that the disordered chain conformation in relaxor ferroelectric polymers remains as the ground state at varied temperatures and applied electric fields, which is in stark contrast to relaxor perovskites whose ground state is strongly dependent on temperatures and external electric fields. The discovery of the absence of thermal‐ and field‐induced phase transition in relaxor ferroelectric polymers makes this class of ferroelectric materials more attractive for advanced electronic and energy applications.

## Graphical abstract

Relaxor ferroelectric polymers exhibit both high dielectric constants and low remnant polarization and thus deliver much higher energy densities and greater charge–discharge efficiencies than normal ferroelectrics for capacitive energy storage applications. This work provides insights into high electrical energy storage properties in relaxor ferroelectric polymers at the molecular level.

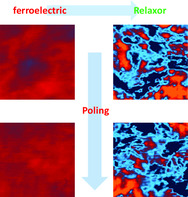

## 1 Introduction

Relaxor ferroelectrics are characterized by a broad maximum in temperature dependence of the dielectric permittivity, whose positions shift to higher temperatures as the frequency of the applied electric field increases.^[^ ^1^, ^2^ ^]^ Relaxor ferroelectrics exhibit unusual properties, such as high and frequency‐dependent dielectric constants over a wide temperature range, ultrahigh piezoelectric response and colossal electrocaloric effect; they are the materials of choice for acoustic sensing, micropositioners, actuators, and electrocaloric cooling devices.^[^ ^3^, ^4^, ^5^, ^6^ ^]^ Different from normal ferroelectrics with long‐range ferroelectric orders, relaxor ferroelectrics are macroscopically paraelectric with no spontaneous polarization at zero electric field, which leads to a very slim polarization–electric field (*P*–*E*) hysteresis loop with nearly zero remnant polarization.^[^ ^7^, ^8^, ^9^ ^]^ Relaxor ferroelectric terpolymers P(VDF‐TrFE‐CFE) and P(VDF‐TrFE‐CTFE) (VDF: vinylidene fluoride; TrFE: trifluoroethylene; CFE: 1,1‐chlorofluoroethylene; CTFE: chlorotrifluoroethylene) exhibit the highest room‐temperature dielectric constants (>50 at 1 kHz) among the known polymers.^[^ ^7^, ^10^, ^11^, ^12^, ^13^ ^]^ The elimination of large polarization hysteresis in P(VDF‐TrFE‐CFE) (58.3/34.2/7.5 mol%) terpolymer in comparison to a typical rectangle hysteresis loop of normal ferroelectric P(VDF‐TrFE) yields a much higher electric energy density with substantially reduced loss.^[^ ^7^ ^]^ The electric energy density of >9 J cm^−3^ obtained in the relaxor terpolymer at 400 MV m^−1^ is greater than those of known unstretched dielectric polymers under the same applied electric field,^[^ ^7^ ^]^ and is comparable to the stretched PVDF homopolymer.^[^ ^14^ ^]^ Moreover, the relaxor terpolymers exhibit a large change in electric displacement with temperature, which gives rise to pronounced electrocaloric effect (e.g., an adiabatic temperature change of 12 °C and an isothermal entropy change of 55 J kg^−1^ K^−1^) near room temperature.^[^ ^9^ ^]^ Because of the large difference in the dimension between the molecular conformations in the polar and nonpolar phases in P(VDF‐TrFE) copolymers, the gradual increase in polarization with the electric field in the relaxor terpolymers produces a giant electrostrictive thickness strain of more than 7%, an elastic energy density of more than 1 J cm^−3^, and an electromechanical conversion efficiency of higher than 30%.^[^ ^10^ ^]^

Although a variety of impressive properties have been achieved in relaxor ferroelectric polymers, research in relaxor polymers is still in its infant stage when compared with their perovskite counterparts.^[^ ^1^, ^2^, ^3^, ^4^, ^5^, ^6^ ^]^ The model of polar nanoregions inside a nonpolar matrix developed from classic lead‐based perovskites, e.g., Pb(Mg_1/3_Nb_2/3_)O_3_‐PbTiO_3_ (PMN‐PT), has been widely used to describe relaxor ferroelectrics.^[^ ^7^, ^10^, ^11^, ^12^, ^13^ ^]^ The current understanding of relaxor behaviors in ferroelectric polymers, such as the hypothesis based on local crystal disorder, is also largely established on the polar nanoregion model.^[^ ^15^, ^16^ ^]^ The defect pinning and nanoconfinement effect was recently proposed to elucidate the polarization and ferroelectric behavior in realxor polymers.^[^ ^17^, ^18^ ^]^ It was postulated that the chemical (irradiation) or structural (CFE or CTFE inclusion) defects act as the pinning source, e.g., physical pinning in P(VDF‐TrFE)‐based terpolymers and chemical pinning in electron‐beam irradiated P(VDF‐TrFE), which can lead to breaking of large ferroelectric domains into nanoscale size and the formation of either polar nanoregions or multidomain structures. More recently, the structural origin of relaxor properties of ferroelectric polymers has been revealed in terms of conformational disorder induced by chain chirality,^[^ ^19^ ^]^ which is very different from perovskite relaxors typically characterized by chemical disorder and sheds new light on the fundamental theory of relaxor ferroelectrics.^[^ ^1^, ^2^ ^]^ The relaxor ferroelectric properties found in pristine P(VDF‐TrFE) and P(VDF‐CTFE) copolymers have been attributed to stem from polytrifluoroethylene (PTrFE) and polychlorofluoroethylene (PCTFE), respectively. The relaxor copolymers are analogues of perovskites PMN‐PT, where PMN is the relaxor and PT is a normal ferroelectric.^[^ ^4^, ^6^ ^]^

In this context, we are motiviated to study phase‐transition behavior under different electric fields^[^ ^20^, ^21^, ^22^, ^23^, ^24^ ^]^ and temperatures^[^ ^25^, ^26^, ^27^, ^28^, ^29^, ^30^ ^]^ in relaxor ferroelectric polymers to elucidate the fundemental difference between peroskite and polymer relaxors. Moreover, the phase stability especially from a microscopic perspective is crucial to practical applications of relaxor ferroelectric polymers. We also focus on the energy storage behavior in relaxor ferroelectric polymers and correlate it with the structure analysis.

## 2 Results and Discussion

### 2.1 Dielectric Energy Storage in Copolymers and Homopolymers

**Figure 1:**
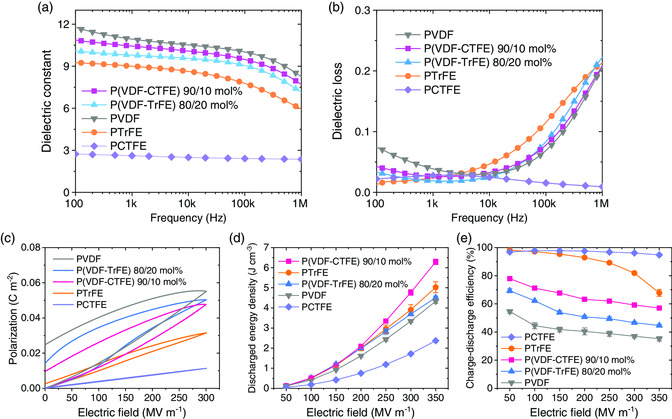
a) Frequency‐dependent dielectric constant measured at room temperature. b) Frequency‐dependent dielectric loss measured at room temperature. c) Unipolar *P*–*E* loops of the polymers at 10 Hz. d) Discharged energy density and e) charge–discharge efficiency. The lines are guides for eyes.

We first examine the effect of electric field on relaxor ferroelectric polymers by examining the dielectric energy storage behavior deduced from the *P*–*E* loops under different electric fields. According to the literature, it remains elusive about the outstanding energy storage performance in terpolymer relaxors achieved by introducing bulky defect introduction (CFE or CTFE) at a concentration of ≈8 mol%,^[^ ^10^, ^11^, ^12^, ^13^ ^]^ whereas the defect content dependence of relaxor properties is unclear. In this regard, the dielectric behavior in relaxor homopolymers, i.e., PTrFE and PCTFE, may be helpful in shedding some insights. **Figure** **1**a,b present the dielectric properties of the polymers as a function of frequency measured at room temperature. The dielectric constant of relaxor PTrFE (≈9 at 1 kHz) is slightly smaller than those of normal ferroelectric P(VDF‐TrFE) 80/20 mol% (≈9.8 at 1 kHz) and relaxor ferroelectric P(VDF‐CTFE) 90/10 mol% (≈10.4 at 1 kHz), whereas PVDF exhibits the largest dielectric response (≈10.9 at 1 kHz) and relaxor PCTFE has the smallest dielectric constant of about ≈2.6 at 1 kHz among the polymers of interest here. The dielectric losses of these polymers remain close to each other, i.e., around 0.02–0.04 at 1 kHz (Figure 1b). The comparison between the dielectric spectra of the copolymers and homopolymers reveals that PTrFE shows a very comparable dielectric response to PVDF. Apparently, the introduction of CTFE co‐monomer into PVDF leads to a considerable decrease in the dielectric constant of the copolymer with a nearly frequency‐independent loss behavior.

The energy storage capability of the ferroelectric polymers was evaluated from the unipolar *P*–*E* loops at room temperature (Figure 1c). It can be seen that relaxor PCTFE shows a remarkably slimmer loop than those of the other polymers. PTrFE also displays slim loops due to its relaxor nature. As shown in Figure 1d, PTrFE exibits higher discharged energy densities than PVDF with the *α* phase (TGTG¯conformation, T: *trans*, G: *gauche*) and ferroelectric P(VDF‐TrFE) 80/20 mol% with the *β* phase (all‐*trans* conformation), e.g., 3.91 versus 3.34 and 3.69 J cm^−3^ at 300 MV m^−1^. The discharged energy density of PTrFE is smaller than that (e.g., 4.77 J cm^−3^ at 300 MV m^−1^) of relaxor P(VDF‐CTFE) 90/10 mol%. The high discharge energy density of seminal P(VDF‐CTFE) 90/10 mol% copolymers (Figure 1d) is thus attributed to its helical conformation rather than TGTG¯ conformation.^[^ ^19^ ^]^ Although PCTFE shows the lowest discharged energy densities among the polymers of interest, its charge–discharge efficiency apparently outperforms all the ferroelectric polymer dielectrics as shown in Figure 1e.

### 2.2 Understanding of Dielectric Behavior in Terpolymers

**Figure 2:**
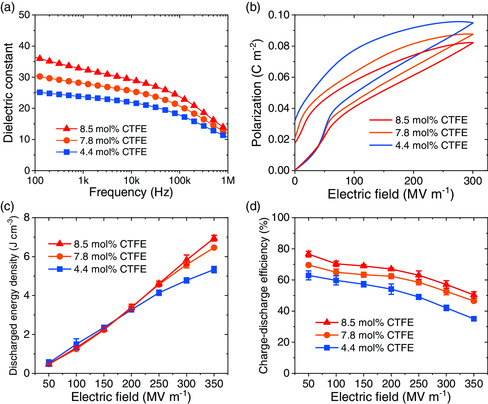
a) Frequency dependence of dielectric constant. b) *P*–*E* loops measured under unipolar electric fields of 10 Hz. c) Discharged energy density. d) Charge–discharge efficiency. The data were measured in P(VDF‐TrFE‐CTFE) terpolymers.

The findings in homopolymers may pave the way for understanding excellent dielectric behavior previously observed in relaxor ferroelectric terpolymers. We synthesized relaxor terpolymer P(VDF‐TrFE‐CTFE)s with variable CTFE concentrations to depict dielectric responses as a function of CTFE inclusion (Figure S1 and S2, Supporting Information). **Figure** **2**a shows the frequnecy‐dependent dielectric constant of the terpolymers measured at room temperature. We find that the dielectric constant of P(VDF‐TrFE‐CTFE) increases with the increase in the CTFE content for the whole frequency range of interest here. In addition, the dielectric loss increases slightly as the CTFE content increases (Figure S3a, Supporting Information).

We evaluate the energy‐storage capability of the terpolymers according to the unipolar *P*–*E* loops (Figure 2b and Figure S3c–e, Supporting Information). It is shown that the maximum polarization decreases and the shape of loops shrinks slightly as the CTFE content increases. As shown in Figure 2c,d, P(VDF‐TrFE‐CTFE) with the highest CTFE content displays the largest discharged energy density and the best charge–discharge efficiency for the defect content of interest here. The enhanced efficiency with the increase in the CTFE content is expected as PCTFE exhibits the highest efficiency (Figure 1d). The increase in the discharge energy density is attributed to the decrease in the remnant polarization due to the nearly zero remnant polarization in PCTFE.

**Figure 3:**
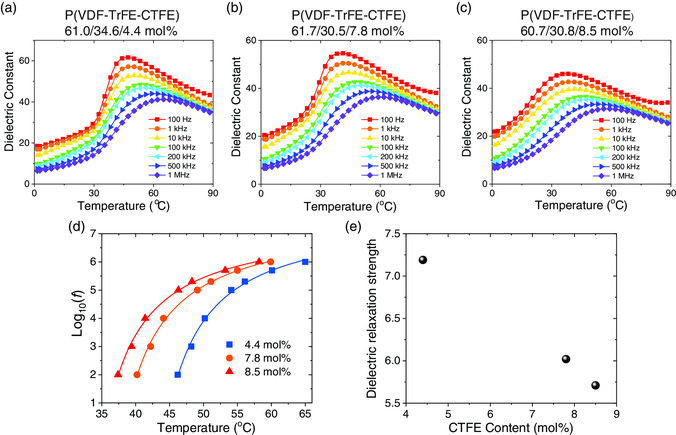
a–c) Temperature dependence of the dielectric constant of P(VDF‐TrFE‐CTFE) with various compositions. d) Logarithm frequency as a function of dielectric peak temperature (heating) for different terpolymers, where fits by the relation ln*f* = ln*f*_0_‐(*T*_0_/*T*_max_)*p* (solid lines) are added. e) DRS *p* versus CTFE content in P(VDF‐TrFE‐CTFE).

To gain further insights into the terpolymer structures associated with the CTFE inclusion, we performed dielectric spectroscopic measurements under different temperatures and frequencies. **Figure** **3**a–c show the dielectric spectra of P(VDF‐TrFE‐CTFE) terpolymers upon heating. Different from dielectric response of normal ferroelectrics, relaxor ferroelectrics typically display the dielectric peaks at the temperature *T*_max_ which depends strongly on the electric field frequency and shifts toward higher temperatures with the increase in frequency. The dielectric response of P(VDF‐TrFE‐CTFE) terpolymers are characterized by relaxor behavior (Figure 3a–c), and the more CTFE inclusion, the broader the shape of dielectric peaks is, which is accompanied by a considerable decrease in the magnitude of the dielectric peak values. As the CTFE content increases, the dielectric peak temperature *T*_max_ shifts toward lower temperature. Meanwhile, the peak temperature for dielectric loss also decreases with the increase in CTFE content (Figure S3B, Supporting Information). We also find that the peak value of dielectric loss remains nearly the same for different CTFE contents. The rapid increase in the low‐frequency dielectric responses near 90 °C shown in Figure 3b,c is extrinsically attributed to ionic conductivity.

The dielectric relaxation of relaxors can be described by many theoretical models. Here, we analyze the relaxor behavior of terpolymers according to the relation ln*f* = ln*f*_0_‐(*T*_0_/*T*_max_)*p*, where *T*_0_ is the equivalent temperature of the activation energy and *p* is a dielectric relaxation strength (DRS).^[^ ^31^, ^32^ ^]^ In this relationship, the parameter can be used to characterize the DRS of relaxors. Specially, normal ferroelectric would have an infinite *p* value. For relaxor, *p* is above 1 and the decrease in *p* indicates a stronger DRS occurring in relaxor ferroelectrics. Figure 3d summarizes that the logarithm frequency versus peak temperature data can be reasonably described by this relation, indicative of a typical relaxor behavior occurring in terpolymers. We find in Figure 3e that the dielectric relaxation becomes stronger in P(VDF‐TrFE‐CTFE) terpolymer as the CTFE content increases. In addition, we carried out electrostrictive strain measurements at both small and large applied electric fields. We find that relaxor terpolymers at low electric fields are nonpiezoelectric (Figure S4, Supporting Information) as the sign of strain shows no change upon the switch in the sign of the applied electric field. The electrostrictive coefficients are usually measured at high electric fields by measuring the electric‐field‐induced strain and polarization response simultaneously (Figure S5a,b, Supporting Information). As a result, we find that the electrostrictive coefficient |*Q*_33_| increases substantially as the CTFE content increases in P(VDF‐TrFE‐CTFE) (Figure S5c,d, Supporting Information). As PCTFE is in a highly disordered helix conformation,^[^ ^19^, ^33^, ^34^ ^]^ the more CTFE introduced, the more disorder the terpolymer will be, which therefore rationalizes the observed experimental results. This argument is also supported by the Fourier‐transform infrared spectroscopy (FTIR) results showing that the characteristic infrared absorbance of the all‐*trans* conformation at around 1288 cm^−1^ shrinks considerably as the CTFE content increases (Figure S6, Supporting Information).

### 2.3 Phase Transition in Relaxor Polymers

**Figure 4:**
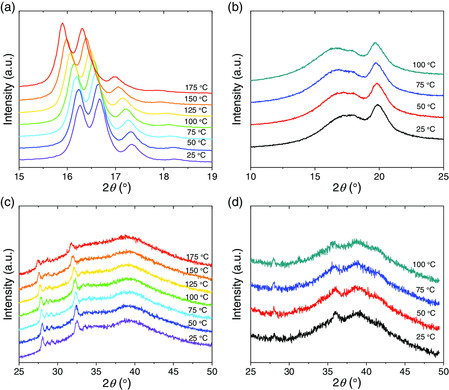
Temperature‐dependent XRD patterns upon cooling: a,b) PCTFE. c,d) P(VDF‐CTFE) 90/10 mol%.

Understanding of structural phase transitions is essential to relaxor ferroelectrics, which remains controversial regarding the phase transitions in relaxor ferroelectric polymers. For instance, previous studies reported the existence of the phase transitions in relaxor ferroelectric polymers based on differential scanning calorimetry (DSC) technique.^[^ ^12^, ^35^, ^36^, ^37^, ^38^ ^]^ We show that P(VDF‐TrFE‐CTFE) terpolymers exhibit no evidence of phase transition according to the temperature dependence of X‐ray diffraction (XRD) and FTIR (Figure S7, Supporting Information), which is in line with the results in P(VDF‐TrFE‐CFE) terpolymer.^[^ ^39^ ^]^ Moreover, we provide structural analysis of the phase transition in newly discovered relaxor polymers based on temperature‐dependent XRD. PCTFE (**Figure** **4**a,b) and P(VDF‐CTFE) copolymers (Figure 4c–f) are newly discovered relaxor polymers.^[^ ^19^ ^]^ Previously, the crystalline structure of P(VDF‐CTFE) was regarded as the *α* phase (TGTG¯conformation, T: *trans*, G: *gauche*).^[^ ^7^ ^]^ However, the recent XRD studies confirm the absence of the characteristic peaks for the *α* phase while atomic force microscopy‐based infrared spectroscopy (AFM‐IR) provides the evidence of conformational disorder in P(VDF‐CTFE).^[^ ^19^ ^]^ As a result, PCTFE and its copolymers P(VDF‐CTFE) are considered to exhibit disordered helix conformation^[^ ^33^, ^34^ ^]^ with their helical period being different from the PTrFE‐based relaxor polymers. The introduction of more VDF monomer into P(VDF‐CTFE) does not necessarily stabilize the *α* phase regardless of temperature because the temperature‐dependent XRD in PCTFE (Figure 3a,b) and P(VDF‐CTFE) copolymers (Figure 4c–f) only exhibit a shift in the pattern upon heating, indicating no phase transition in PCTFE and P(VDF‐CTFE). As a result, the disordered helix conformation in both PCTFE and P(VDF‐CTFE) remains in the ground state from room temperature to high temperatures that are close to the melting temperature. To rule out the contribution of glass transition to the relaxor behavior in the P(VDF‐CTFE) copolymer, we estimate its glass‐transition temperature *T*_g_ as −32 °C using the linear approximation (*T*_g_ = −40 °C, PVDF; *T*_g_ = 45 °C, PCTFE). In this regard, *T*_g_ in P(VDF‐CTFE) is well below the dielectric peak temperature *T*_max_ = 15 °C (ref. [19]). As a result, the glass transition may play a negligible role in affecting the dielectric relaxations in relaxor P(VDF‐CTFE) near *T*_max_.

**Figure 5:**
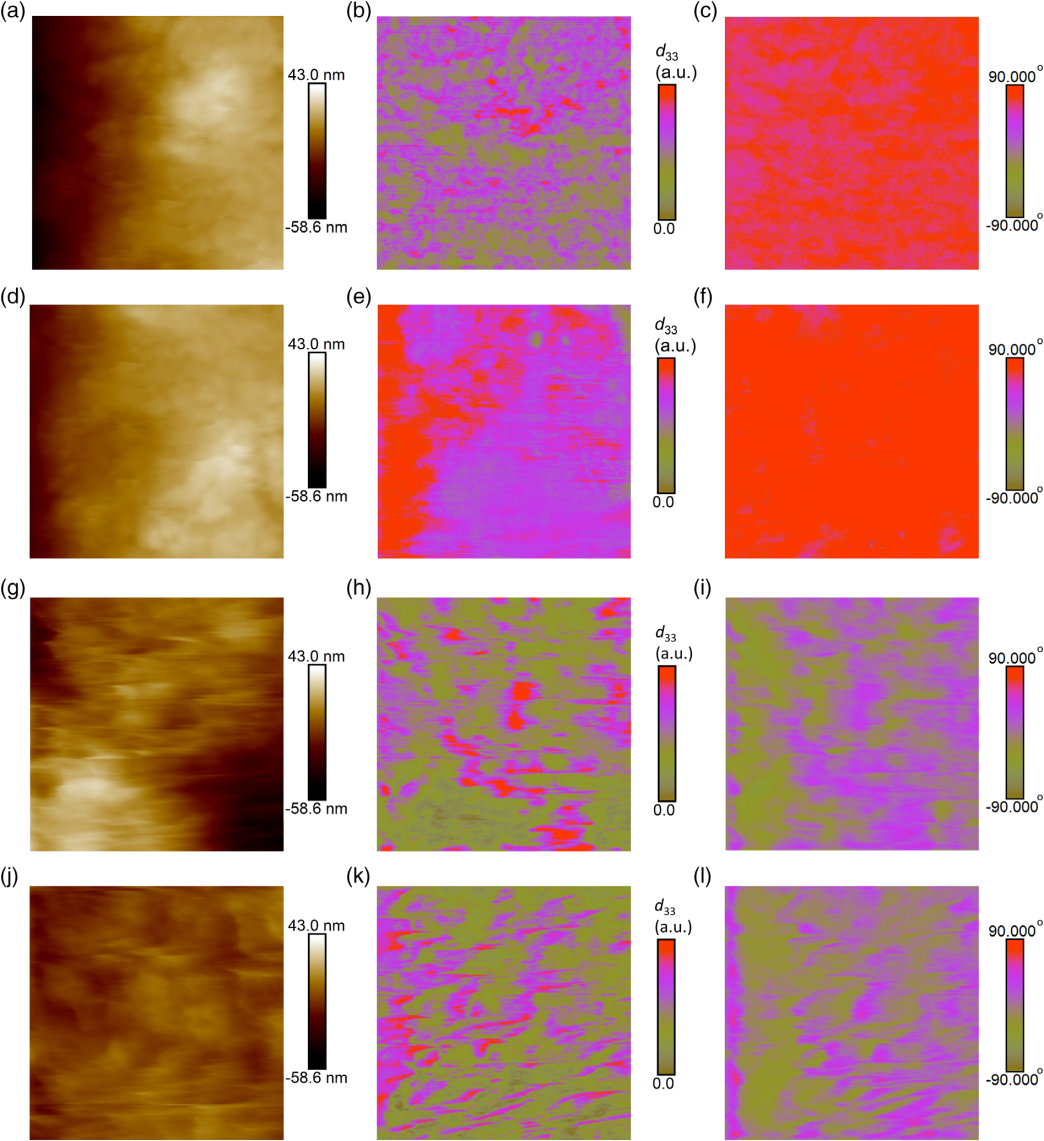
a,d) Topography (1 × 1 μm^2^). b,e) PFM amplitude. c,f) PFM phase in P(VDF‐TrFE) 80/20 mol%. The upper panel is under zero field. The bottom panel is polarized at 100 MV m^−1^ for 10 min at room temperature. g,j) Topography (1 × 1 μm^2^). h,k) PFM amplitude. i,l) PFM phase PTrFE. The upper panel is under zero field. The bottom panel is polarized at 100 MV m^−1^ for 10 min at room temperature.

Piezoresponse force microscopy (PFM) is useful for studying electric‐field and mechanical stress‐induced polarization switching in nanoscale thin films.^[^ ^40^, ^41^, ^42^ ^]^ We use PFM to evaluate the electric field‐induced phase transition in ferroelectric polymers. As our film thickness is thick (about 15 μm), here we polarize the bulk film with metallized biaxially‐oriented polypropylene (BOPP) films as electrodes (DC, 100 MV m^−1^, 5 min). According to the phase diagram of P(VDF‐TrFE) copolymers,^[^ ^43^ ^]^ we choose a typical composition at high VDF contents, i.e., P(VDF‐TrFE) 80/20 mol% which is typically ferroelectric. We show the PFM data in this ferroelectric composition in **Figure** **5**. We observe that the application of an electric field of 100 MV m^−1^ leads to a large enhancement of piezoelectric response (Figure 5b,e) with a monodomain structure (Figure 5c,f). Moreover, both PFM amplitude and phase images in relaxor PTrFE show no noticeable difference without (Figure 5h,i) and with the poling field (Figure 5k,l), which indicates the absence of the phase transition from relaxor to ferroelectric phases induced by external electric fields.^[^ ^39^ ^]^ These results demonstrate that the long‐range ferroelectric single domain can be achieved in the ferroelectric composition by the application of external fields, whereas the short‐range relaxor state remains in the relaxor composition even under a large electric field. This result is in line with the previous electric‐field‐induced strain measurements showing typical electrostriction behavior up to 200 MV m^−1^ in PTrFE.^[^ ^19^ ^]^ As a result, the conformational disorder may be the energetically preferred state, which requires a high energy (much higher electric field than 100 MV m^−1^ used here) to approach the ordered ferroelectric phase.

**Figure 6:**
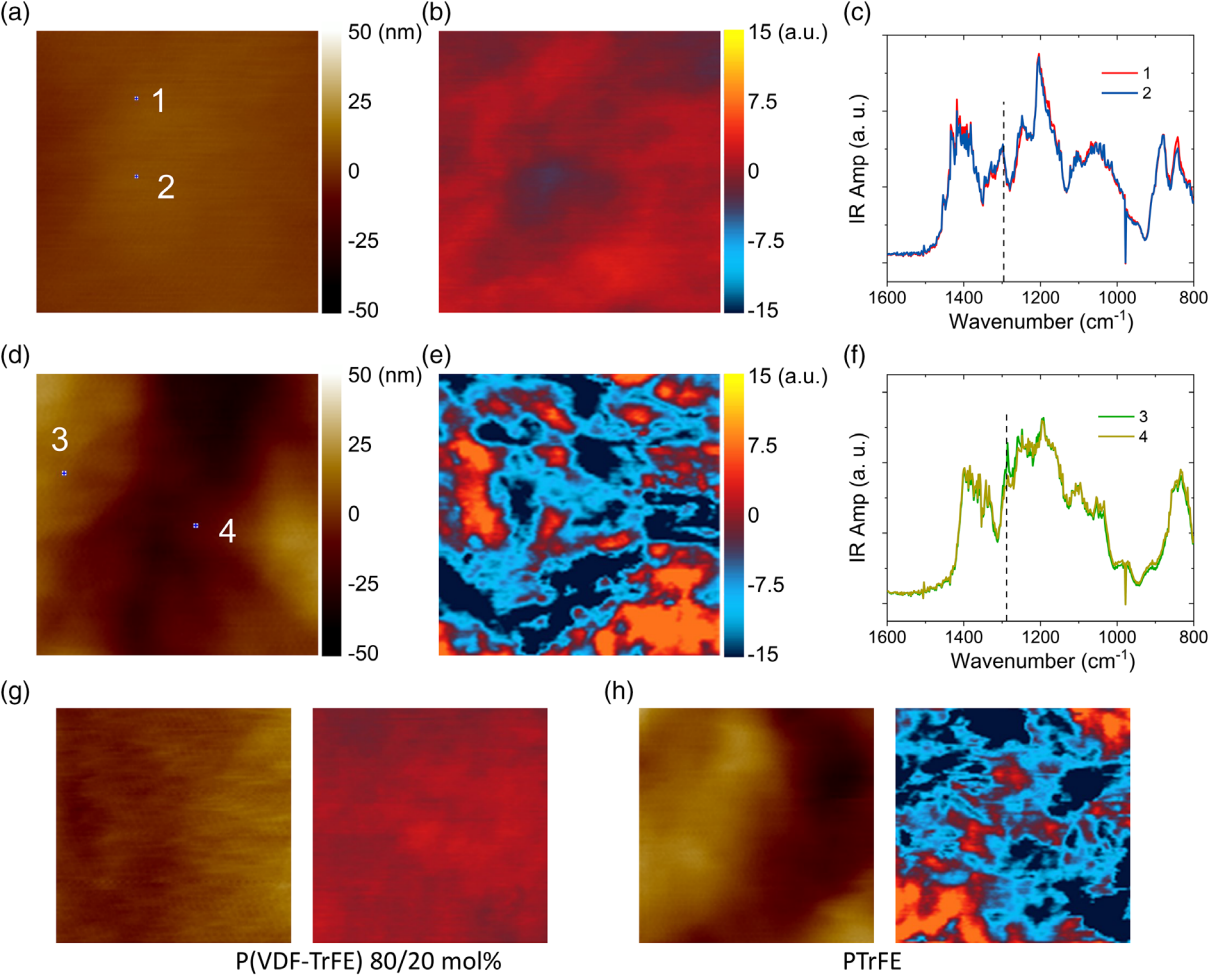
a,d) Simultaneously measured topography and b,e) AFM‐IR chemical map at 1288 cm^−1^. The size of (a–e) is 1 × 1  μm^2^. c,f) Local spectra of the positions marked in (a) and (d). The upper panels (a–c) are P(VDF‐TrFE) 80/20 mol%. The middle panels (d–f) are PTrFE. g,h) Simultaneously measured topography and AFM‐IR chemical map in poled P(VDF‐TrFE) 80/20 mol% and poled PTrFE, respectively.

We finally use AFM‐IR to gain deeper structural insights into the field‐induced behavior by PFM. Here we use contact mode^[^ ^44^ ^]^ which is the same as PFM but different from the tapping mode used in our previous AFM‐IR measurements.^[^ ^19^ ^]^ The advantage of the contact mode in AFM‐IR over the tapping mode is a better signal‐to‐noise (S/N) ratio of local spectra due to the fact that the contact probe is not tapping/hammering the surface of the fixed local area during operation and the signal is amplified by measurement at the contact resonance. Such tapping/hammering motion between probe and sample at a fixed location can deliver mechanical energy and alter the local surface, causing fluctuation of the contact resonance and therefore lowering the S/N ratio.^[^ ^45^ ^]^ Such probe‐sample interaction can be tackled using analytical methods,^[^ ^46^ ^]^ but has not been understood fully. Interestingly, we find that the existence of conformational disorder in PTrFE is regardless of the AFM‐IR mode. The results by the contact AFM‐IR mode are consistent with our previous findings.^[^ ^19^ ^]^ For instance, a uniform chemical pattern is found in normal ferroelectric composition P(VDF‐TrFE) 80/20 mol% (**Figure** **6**a,b), in contrast to that of PTrFE which exhibits a strong spatial dependence due to the conformational disorder (Figure 6d,e). Our results reveal the existence of conformational disorder in terms of large change in the local spectra (Figure 6c,f), which was also reported in tapping mode AFM‐IR.^[^ ^19^ ^]^ Moreover, the AFM‐IR results on the poled samples show that the chemical pattern of P(VDF‐TrFE) 80/20 mol% becomes more uniform compared with the unpoled samples (Figure 6g) while it remains nearly unchanged in PTrFE (Figure 6h). These results not only rationalize the PFM results but also indicate a very large energy barrier to convert the conformational disordered phase into ordered phase in relaxor ferroelectric polymers which differs significantly from the perovskite counterparts.^[^ ^20^, ^21^ ^]^

## 3 Conclusion

In summary, we have demonstrated the absence of phase transition in relaxor ferroelectric polymers upon thermal and electric changes, which indicates that the conformational disorder inherent to relaxor polymers remains in the ground state. We observe that relaxor homopolymer exhibits very high charge–discharge efficiencies, which helps to rationalize the excellent capacitive performance observed in relaxor ferroelectric terpolymers. Our insights into relaxor ferroelectric polymers at the molecular level may provide a basis for the design of high‐performance functional materials for advanced electronic and energy devices.

## 4 Experimental Section

4.1

4.1.1

##### Sample Preparation

Polymer powders with various compositions were synthesized via suspension polymerization. Nuclear magnetic resonance (NMR) spectra were obtained by a Bruker CDPX‐300 spectrometer (300 MHz). The sovent used is acetonitrile‐*d*_3_ (VWR). Freestanding polymer films were prepared via solution‐casting approach. The typical thickness of the films is about 15 μm.

##### Characterization

XRD *θ*–2*θ* scans were acquired using a PANalytical X'pert Pro MPD diffractometer in the Bragg‐Brentano geometry with a source emitting both Cu Kα1 and Cu Kα2 (wavelength *λ*_Kα1_ = 1.54059 Å, *λ*_Kα2_ = 1.54442 Å). A high‐temperature stage was used in temperature‐dependent XRD measurement. A silicon substrate was added between the polymer and the stage. The temperature was controlled by changing the voltage across a thermal resistance. The cooling ramp rates was 1 °C min^−1^. After the system reached the target temperature, the temperature was held for 10 min to maintain the equilibrium state. Then the measurement was performed.

Gold electrodes with a typical thickness of 60 nm and a diameter of 3 mm were sputtered (Denton Vacuum, Desk IV) on both sides of the polymer films for the electrical measurements. Dielectric spectra were acquired using dielectric E4980A precision LCR meter (Keysight) from 100 Hz to 1 MHz. To measure unipolar *P*–*E* field loops, a Sawyer–Tower circuit was used, where the films with electrodes were subjected to a triangular bipolar wave. The PFM measurements were performed using an AFM (Bruker Icon) in contact mode. The AFM‐IR measurements were performed on a NanoIR2 system (Bruker) operating with top‐down illumination using a gold‐coated silicon nitride probe (0.07–0.4 N m^−1^ spring constant, 13 ± 4 kHz resonant frequency, Bruker). The tunable QCL IR laser illuminated samples with repetition rate tuned to match the contact resonance frequency on the sample‐probe contact interface. All spectra were collected at 2 cm^−1^ and were averaged eight times with 128 coeverages for each data points. For AFM‐IR mapping, the scan rate was set to 1 Hz with 256 pt resolution.

## Conflict of Interest

The authors declare no conflict of interest.

## Data Availability Statement

Research data are not shared.

## Supporting information

Supplementary Material
