# Supplementary material for: Relaxor Ferroelectric Polymers: Insight into High Electrical Energy Storage Properties from a Molecular Perspective
Source: Small Sci. 2021 Feb 15;1(3):2000061. doi: 10.1002/smsc.202000061 (PMC11935794; doi:10.1002/smsc.202000061)
Supplement: Supplementary file 1 — Supplementary Material [file SMSC-1-2000061-s001.pdf]

Copyright WILEY-VCH Verlag GmbH & Co. KGaA, 69469 Weinheim, Germany, 2018.

Supporting Information

Relaxor ferroelectric polymers: insight into high electrical energy storage properties from a molecular perspective

*Yang Liu, Yen-Ting Lin, Aziguli Haibibu, Wenhan Xu, Yao Zhou, Li Li, Seong H. Kim and Qing Wang**

Dr. Y. Liu, A. Haibibu, Dr. W. Xu, Dr. Y. Zhou, L. Li, Prof. Q. Wang
Department of Materials Science and Engineering, The Pennsylvania State University, University Park, Pennsylvania 16802, USA
E-mail: wang@matse.psu.edu

Y.-T. Lin, Prof. S. H. Kim
Department of Chemical Engineering, The Pennsylvania State University, University Park, PA 16802, United States

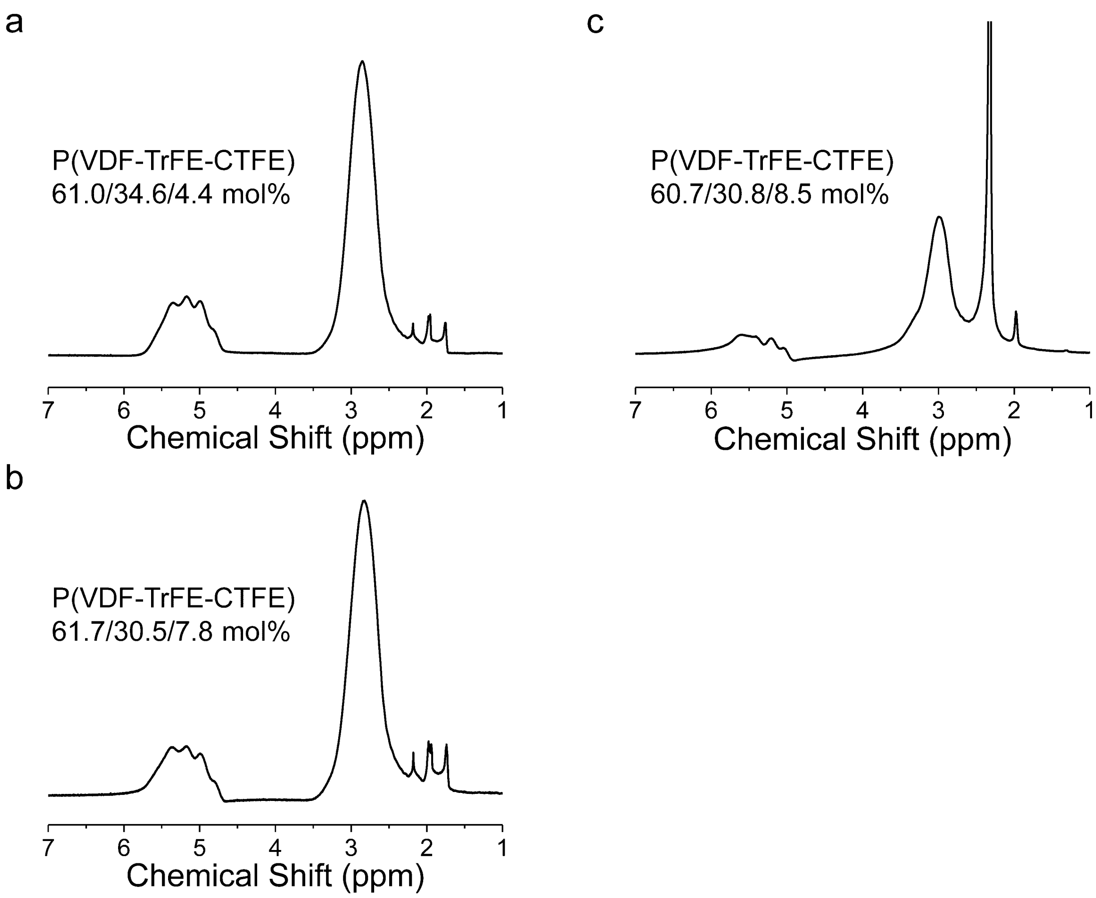

Figure S1. (**a**)-(**c**) ^{1}H Nuclear magnetic resonance (NMR) spectra of terpolymers with different compositions.

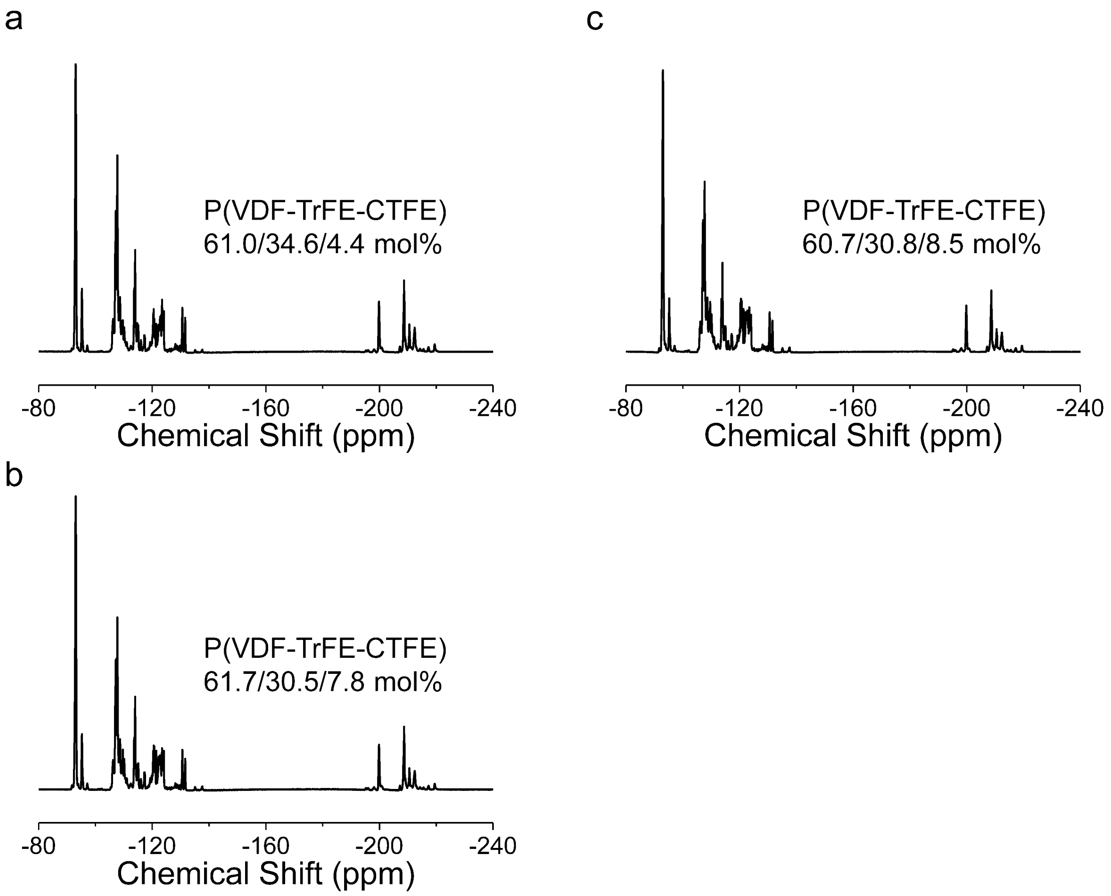

Figure S2. (**a**)-(**c**) ^{19}F NMR spectra of terpolymers with different compositions.

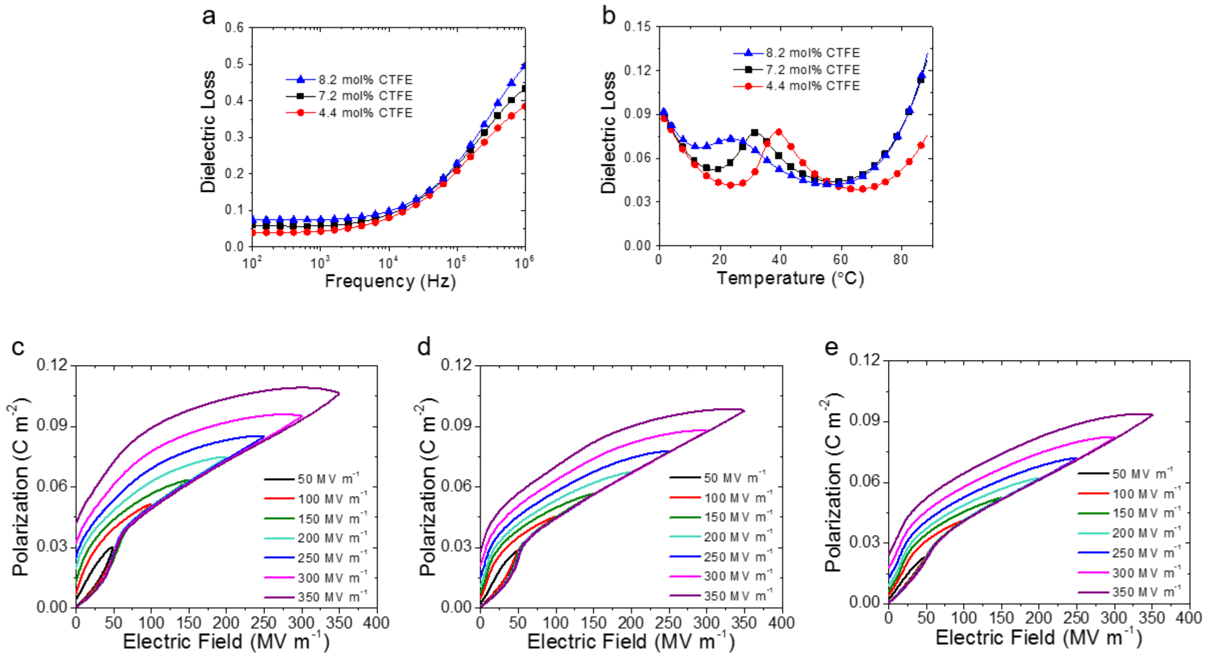

Figure S3. (**a**) Dielectric loss as a function of frequency at room temperature. (**b**) Temperature dependence of dielectric loss at 1 kHz. (**c**)-(**d**) Unipolar polarization-*electric* field loops (10 Hz) in terpolymers with different compositions.

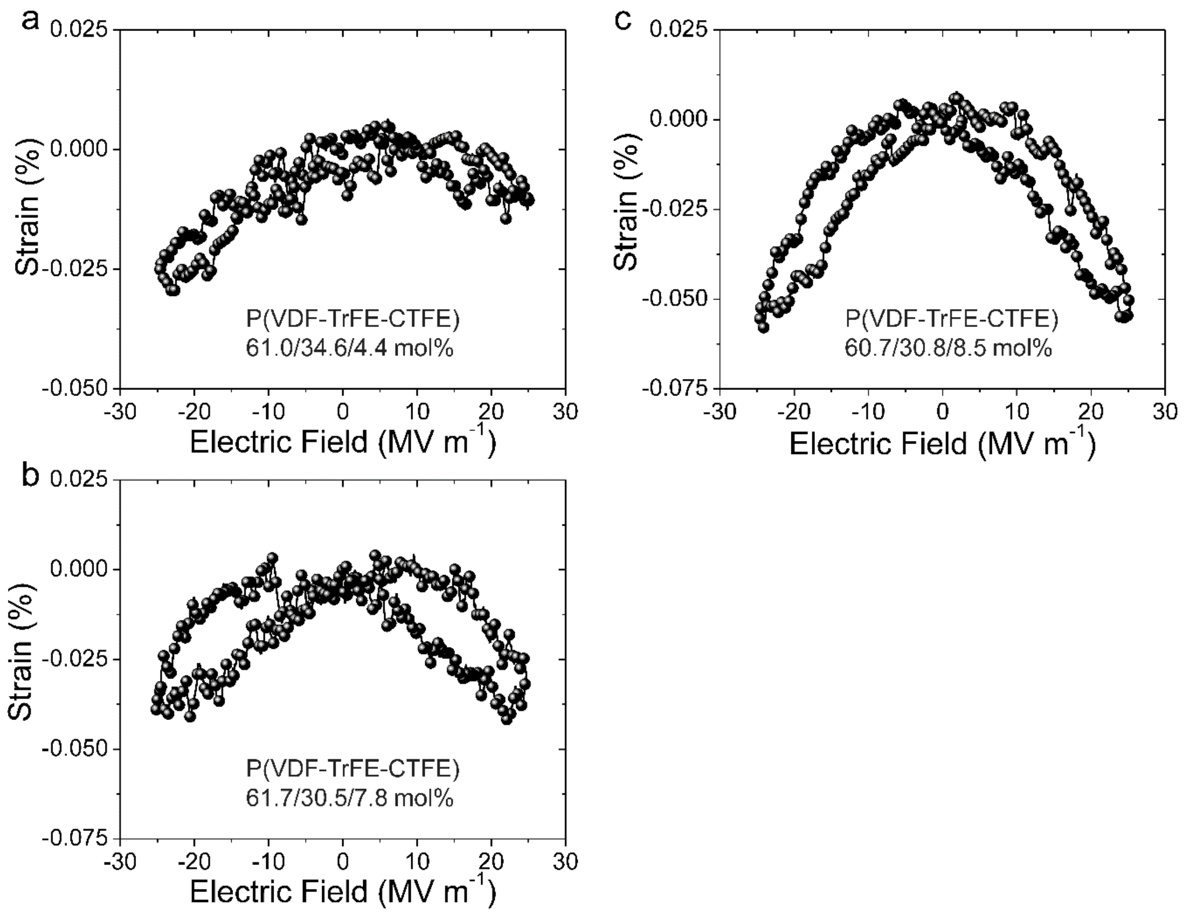

Figure S4. Electric-field induced strain at low fields for different terpolymers.

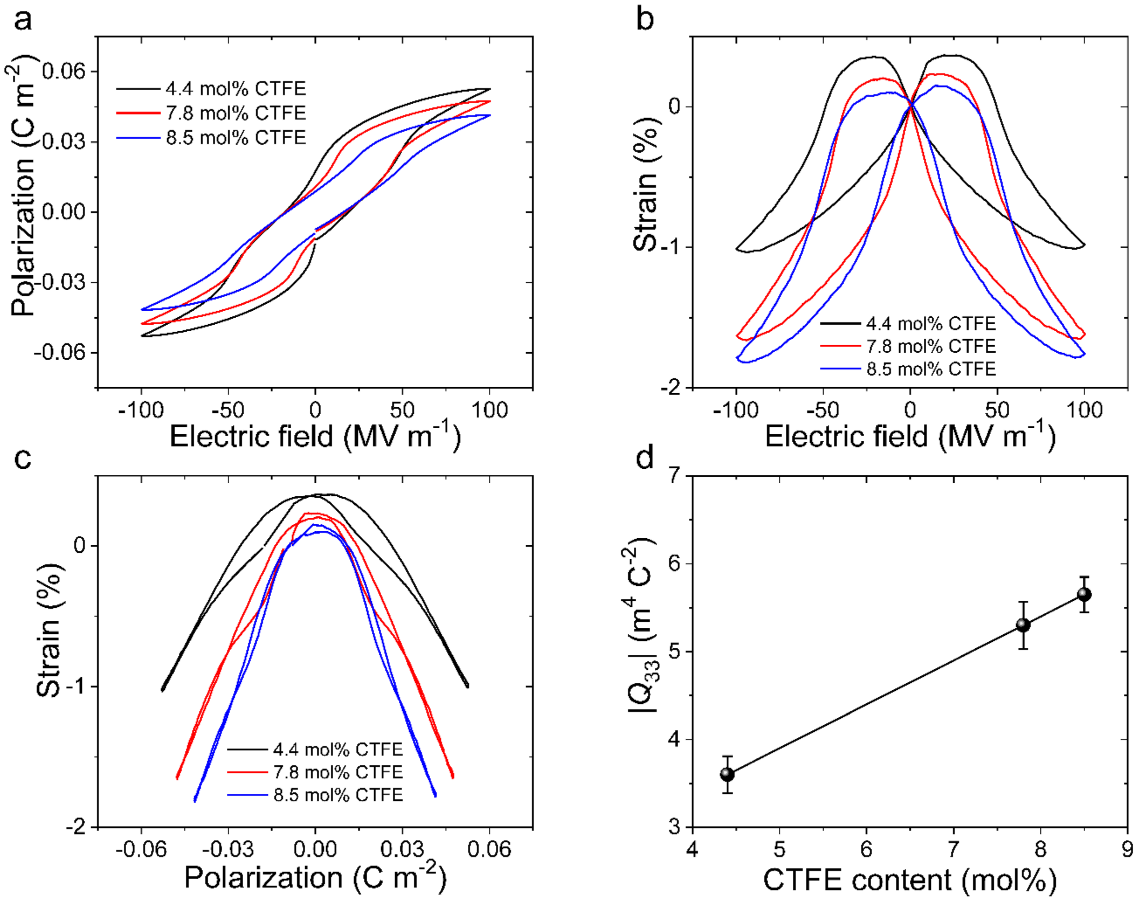

Figure S5. (a) *P-E* loops measured under bipolar electric fields of 1 Hz. (b) Electric-field induced strain. (c) Strain as a function of polarization. (d) Electrostictive coefficients.

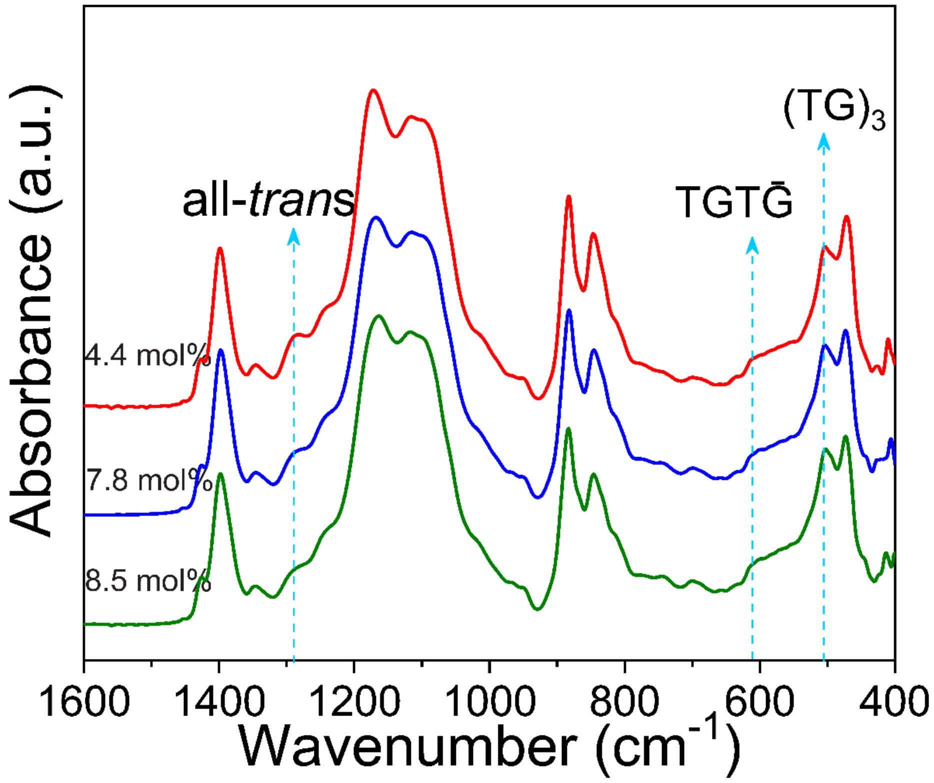

Figure S6. FTIR results.

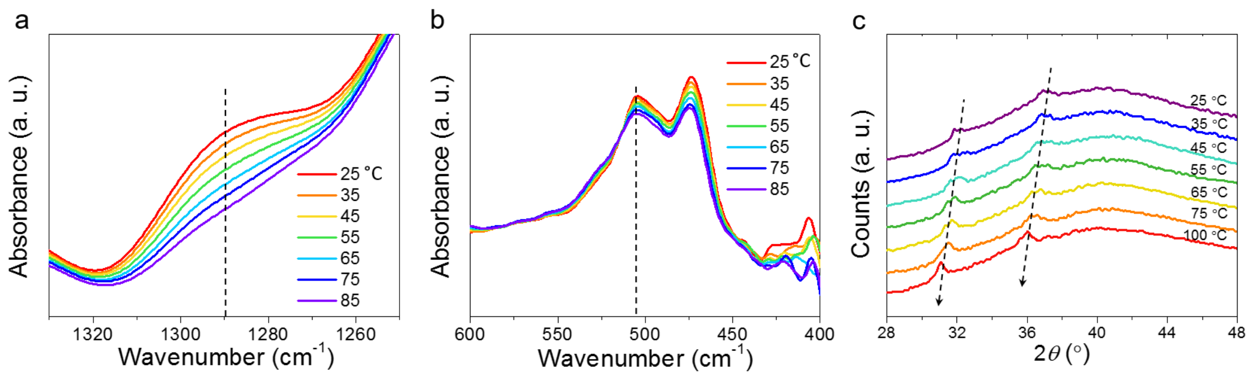

Figure S7. Infrared absorbance bands of P(VDF-TrFE-CTFE) 61.7/30.5/7.8 mol% terpolymer measured upon heating. The dashed lines indicate the characteristic bands for all-*trans* and 3/1-helix conformations at around (a) 1290 cm^{-1} and (b) 507 cm^{-1} . Temperatur dependence of XRD upon heating.
